# Life Capital

**Published:** 1901-01-05

**Authors:** 


### "LIFE CAPITAL."

To those who are fond of figures and of the often puzzling problems which are presented by statistics there is much that is of interest in the tables given by Mr Shirley Murphy, Medical Officer of Health to the County of London, in the annual report which he has just issued. Many interesting speculations, for example, might be founded on the facts he gives regarding the curious variations of the marriage rate from year to year, while the gloomiest prognostications might be drawn, by those who incline to pessimism, from the steady and almost unbroken diminution of the birth rate during the past quarter of a century. Let us, however, concentrate our attention upon the death rate. If other things were to remain the same, it is obvious that the ratio of the deaths to the population in any community ought to give a fair basis on which to judge of its healthiness and to estimate the average prospects of life offered to every infant born to it. No sooner, however, do we penetrate below the mere surface of things than we find that a multitude of circumstances besides mere healthiness influence the mortality in a district, and that the most erroneous conclusions may be arrived at by a casual comparison of crude death rates. The point to which we would just now specially direct attention is one which is insisted on by Mr. Shirley Murphy in regard to what he speaks of as "life capital," namely, that death rates tell us little unless we know the sort of people who die, and that in estimating the influence of any variation of its death rate upon a community we must not merely count the number of the dead, but must carefully consider what prospects of life they would have had if disease or accident had not carried them off, so that we may estimate the loss of "life capital" to the community involved by their decease. Putting the matter in that peculiarly impersonal manner which statisticians love, it is clear that a very considerable increase in the number of deaths among those whose life's work is done—say those between seventy and eighty years of age—might not only be no loss, but might even be an actual gain to a community by relieving it of the burden of supporting so many non-producers. The death of a child, however—a child which has within it a potentiality of many years of active productive life—is quite another affair; while the death of a young person who has weathered all the accidents of childhood, and is just beginning to repay the trouble spent in rearing and education, is a dead loss from an economic point of view. If, then, we are in any way to understand the bearing of any variation in the death rate upon the prosperity of a community we must express our losses, not in numbers of deaths, but in quantity of "life capital" destroyed.

What Mr. Shirley Murphy has done is first of all to construct a "life table" for London from the accumulated statistics collected during the ten years 1881-90. In this table the whole population is divided into twelve age-groups, and the mean future life—in other words the prospect of life—is calculated for each age-group, and this is done for males and females separately; a matter of some importance, seeing that the females all through have a prospect of longer life than the males of the same age-group. Having, then, got his "life table" he has sorted out all the deaths in London according to sex and age, and for each year since 1891, and applying the "table" to each group, he has been able to show how far we are gaining or losing, improving upon or falling back from what we may call the "standard" of 1881-90, and to express this not merely in number of lives, but in number of years of "life capital" saved or lost in comparison with the standard.

From this it appears that the year 1897 was the heathiest year of those under consideration, the number of lives saved, as compared with the standard, being 10,336, while the amount of years of "life capital" saved was 367,815. Since then we have gone back considerably, the lives gained in the year 1899 being only 3,568, while the "life capital" gained was but 264,297. Now here comes an interesting thing, and a thing which shows the importance of working out the statistics in a careful manner. At first sight a drop from ten thousand to three thousand in the number of lives gained appears a frightful fall; but on examination it appears that the year 1899 was almost as good as the year 1897, both in regard to lives saved and "life capital" saved among people under 25 years of age. Almost the whole loss occurred in the older people, commencing in men at the group over 35, and in women at the group over 45.

Now if we turn to particular diseases we find that the death rates for the different years are divided into those for people below and above 25 years of age; but the life table has not been applied to the individual diseases. Nevertheless, we find some interesting facts. There are certain diseases in regard to which we were distinctly worse off in 1899 than in the "standard" period; for instance, diphtheria, diarrhœa, cancer, and influenza. The whole of the increased loss of life from diphtheria fell upon the young; there is no difference at all among those above 25. With diarrhœa, again, not only does all the increase fall upon the young, but even more, for among those above 25 years of age there is an actual improvement, all of which is neutralised by the increased mortality from this disease among the young. Thus we have in diphtheria and diarrhœa two diseases against which we must place a very heavy loss of "life capital," for they kill those who have their whole working life before them. In regard to cancer the boot is on the other leg. Both in old and young cancer has increased; but the increase is twenty-five times as great in the older group as it is in the younger, so that the drain of this disease, great as it is upon "lives," is not so great upon "life capital."

Statistics are not generally very interesting things, and the errors to which they are open are very great; nevertheless if figures are to be of any use in municipal life, and if we are to obtain from them any safe guidance, they must be worked out like these, carefully and laboriously, and we may fairly point out that the annual report of the Medical Officer to the County of London is a document which, from a statistical point of view, does the highest credit to Mr. Shirley Murphy and to the staff by whom he has been assisted.

